# Evolutionary Importance of the Intramolecular Pathways of Hydrolysis of Phosphate Ester Mixed Anhydrides with Amino Acids and Peptides

**DOI:** 10.1038/srep07440

**Published:** 2014-12-11

**Authors:** Ziwei Liu, Damien Beaufils, Jean-Christophe Rossi, Robert Pascal

**Affiliations:** 1 Institut des Biomolécules Max Mousseron, UMR5247 CNRS – University of Montpellier

## Abstract

Aminoacyl adenylates (aa-AMPs) constitute essential intermediates of protein biosynthesis. Their polymerization in aqueous solution has often been claimed as a potential route to abiotic peptides in spite of a highly efficient CO_2_-promoted pathway of hydrolysis. Here we investigate the efficiency and relevance of this frequently overlooked pathway from model amino acid phosphate mixed anhydrides including aa-AMPs. Its predominance was demonstrated at CO_2_ concentrations matching that of physiological fluids or that of the present-day ocean, making a direct polymerization pathway unlikely. By contrast, the occurrence of the CO_2_-promoted pathway was observed to increase the efficiency of peptide bond formation owing to the high reactivity of the *N*-carboxyanhydride (NCA) intermediate. Even considering CO_2_ concentrations in early Earth liquid environments equivalent to present levels, mixed anhydrides would have polymerized predominantly through NCAs. The issue of a potential involvement of NCAs as biochemical metabolites could even be raised. The formation of peptide–phosphate mixed anhydrides from 5(4*H*)-oxazolones (transiently formed through prebiotically relevant peptide activation pathways) was also observed as well as the occurrence of the reverse cyclization process in the reactions of these mixed anhydrides. These processes constitute the core of a reaction network that could potentially have evolved towards the emergence of translation.

The biosynthesis of peptides involves aminoacyl adenylates (aa-AMPs), formed through the reaction of ATP with α-amino acids (aas) (Fig. 1), that are subsequently used to aminoacylate tRNA. Their standard free energy of hydrolysis value Δ*G*°′ = *ca*. −70 kJ mol^−1^, determined for Tyr-AMP^1^, ranks them among the energy-richest biochemicals. Aa-AMPs possess a phosphate group transfer potential much higher than ATP^1^ and might then constitute adenylating agents as well as aminoacylating agents^2^,^3^. The otherwise unfavourable^1^ reaction of ATP with α-amino acids (*K* = 3.5 × 10^−7^) is driven towards completion by selective stabilization of aa-AMPs in the active sites of aminoacyl tRNA synthetases (aaRSs). They usually remain sequestrated by the enzyme and are not released in solution before reacting with tRNA. The importance of this process can be appreciated by considering that the set of aaRS enzymes, responsible for the association of amino acids with their cognate tRNAs, actually holds the key of the genetic code. The evolutionary path through which adenylates were introduced in the process remains unidentified. In addition of being thermodynamically unfavourable, the spontaneous reaction is indeed very slow in the absence of enzyme^4^,^5^, so that the emergence of the biochemical amino acid activation pathway remains unexplained before a set of catalysts (very probably ribozymes) could lead to an embryo of the genetic code for prebiotically available amino acids^6^. In spite of this obstacle, the evolution of this pathway from an abiotic process of random peptide formation *via* the polymerization of α-amino acid mixed anhydrides with phosphate (aa-PMAs) or phosphate esters (aa-PEMAs) and adenylates (aa-AMPs) has prompted much work^7^,^8^,^9^,^10^. However, the abiotic formation of adenylates or their analogues from phosphate anhydrides did not receive any experimental support. As a matter of fact, the claim^11^ that ATP is capable of driving the polymerization of α-amino acids on clays through aa-AMP intermediates turned out to be non-reproducible^12^. Though the genetic code might have evolved late in the hypothesis of an “RNA world” without needing ATP activation as shown by the successful selection of ribozymes capable of aminoacylating RNAs using either amino acid esters^13^ or activated RNAs^14^, an early co-evolution involving the chemistries of nucleotides and amino acids is consistent with the comparatively higher abundance of the latter as the products of abiotic processes. Therefore, selecting the co-evolutionary option, the elucidation of the potential evolutionary process through which aa-AMPs could have been introduced requires the identification of simple pathways capable of leading to these intermediates. A likely possibility is the reaction of α-amino acid *N*-carboxyanhydrides (NCAs) with inorganic phosphate^15^ and its esters including adenylates that takes place spontaneously at moderate pH^16^,^17^ (Fig. 2a). This possibility is supported by the role of NCAs deduced from the literature^2^ and the disclosure of realistic abiotic pathways for their formation during the last decade^18^,^19^. Since the activation of the C-terminus in peptides has recently been identified as a plausible prebiotic pathway and involves the formation of 5(4*H*)-oxazolone intermediates^20^, it is reasonable that similar mixed anhydrides with phosphates involving acylated amino acids (acyl-aa-PEMAs) or peptides (peptidyl-PEMAs) could be formed by reaction of the energy-rich cyclic intermediate (Fig. 2b). The occurrence of abiotic pathways leading to aa-PEMA or peptidyl-PEMA must have preceded their involvement in chemical evolution. However, the low stability of these mixed anhydrides and the availability of highly reactive cyclic intermediates prone to polymerize more easily renders their role in early abiotic processes of peptide formation highly questionable.

The kinetic stability of aa-AMPs and of other aa-PEMAs has been studied in aqueous solution leading to contradictory results in the literature^21^,^22^,^23^,^24^. Of particular interest with regard to an evolutionary context is the description of a highly efficient CO_2_-catalyzed path of hydrolysis^21^,^22^,^23^. No definitive mechanism has been proposed but the intermediacy of NCAs is highly probable^2^,^25^,^26^ since other activated amino acids (nitrophenyl esters, thioesters) proved to undergo conversion into NCAs in hydrogen carbonate buffers^25^. This analysis casts doubts on the possibility that aa-AMPs constitute efficient monomers for the abiotic formation of peptides in aqueous solutions^2^,^3^,^26^ since most early Earth aqueous environments are likely to have contained CO_2_ or HCO_3_^−^. The present investigations were aimed at providing data on the efficiency of the CO_2_-promoted pathway (Fig. 3a) in aqueous solution at neutral pH and in the presence CO_2_ concentrations compatible with early Earth environments and at clearly identifying the NCA as an intermediate. They address both the issues of the stability of aa-AMPs and of other aa-PEMAs and that of the path of peptide formation. They demonstrate the prevalence of the CO_2_-promoted pathway in the hydrolysis of adenylates. More importantly, using model amino amide reactants, they additionally demonstrate that peptide bond formation takes place predominantly from the cyclic intermediates rather than directly from the mixed anhydrides ruling out any possibility of considering the latter as direct peptide precursors at early stages of chemical or biochemical evolution. Lastly, considering NCAs as likely precursors of aa-AMPs and aa-PEMAs, the hypothesis of an abiotic formation of non-coded peptides through these mixed anhydrides becomes unnecessary. The evolution of translation must then have proceeded through a pathway independent from abiotic polymerization. This work also addresses the more general goal of understanding the stability of phosphate mixed anhydrides of amino acids and peptides in aqueous media at moderate pH. As a matter of fact, though *N*-acylation is an obvious way to prevent CO_2_ participation, another intramolecular path of breakdown through 5(*4H*)-oxazolones is possible in the case of acyl-aa-PEMAs (Fig. 3b). Therefore, the issues of the importance of the NCA and 5(*4H*)-oxazolone pathways in the reactions of the corresponding mixed anhydrides (Fig. 3) are raised as well as that of the potential role of these cyclic intermediates as potential prebiotic precursors of these mixed anhydrides (Fig. 2). The consequences of these chemical pathways as factors determining early biological evolution of amino acid activation processes and their constraints on the contemporary biochemistry of adenylates will also be discussed.

## Results

Experiments were carried out from model systems derived from *O*-methylated tyrosine **5** (Fig. 4) likely to be representative of the reactivity of usual amino acid derivatives. The UV-absorption of the tyrosine side chain (*λ*_max_ = 273 nm) was selected to monitor reactions by HPLC at a reasonably low (0.05–1 mM) concentration range in which activated intermediates have a lifetime sufficient for their behaviour to be determined. Furthermore, phenol methylation was introduced to simplify analyses by avoiding any side-reaction of this group. Reactions were carried out in non-nucleophilic MES or MOPS buffers at pH values of 6.5 or 7.5, respectively, whereas 50 mM phosphate or methyl phosphate buffers were used for studying the transient formation of mixed anhydrides. Analyses were performed to monitor the reaction progress of samples stored in the HPLC systems located in a room maintained at the temperature of 20°C. Fast reactions were monitored by withdrawing 1 mL samples from the reaction medium and the reaction was blocked by addition of a formic acid solution to bring the pH to a value below 4 (Supplementary information).

### NCAs as intermediates of aa-PEMA reactions promoted by CO_2_

The hydrolysis of methyl phosphate mixed anhydride **1b** was studied in buffered solutions in the presence of varying contents of CO_2_/HCO_3_^−^. The reaction rates were observed to strongly depend on the presence of CO_2_ as shown by a *c.a.* 4 fold increase in rate using pH 6.5 MES buffers previously equilibrated with air as compared with a solution flushed with N_2_ for 60 min (Fig. 5, panel A). The rates could be reduced by further c.a. 35% by extensive degasification through cycles of freezing at −95°C/gas removal under vacuum/melting in a closed vessel. Under the conditions of the experiment displayed in the panel A of Fig. 5, the starting material **1b** (HPLC retention time, r.t. 4.6 min, method A) disappeared slowly and several species containing the methoxyphenyl moiety (*λ*_max_ 273 nm) were observed, namely the free amino acid **5** (r.t. 8.4 min) representing the main product of hydrolysis but also several peaks corresponding to the dipeptide H-Tyr(Me)-Tyr(Me)-OH (r.t. 22.7 min) and the diketopiperazine *cyclo*-Tyr(Me)-Tyr(Me) (r.t. 23.6 min), very probably resulting of the cyclization of the mixed anhydride H-Tyr(Me)-Tyr(Me)-OPO_3_Me^−^, which has not been properly identified. The presence of these two products was confirmed by HPLC-MS analysis ([M + H] = 373.2 at r.t. 1.52 min and 355.2 at r.t. 1.88 min, method C). By contrast, the addition of 2 or 10 mM NaHCO_3_ to the buffer led to the fast disappearance (≤1% after 3 min) of the mixed anhydride **1b** as monitored by HPLC analysis (Fig. 5, panel B). An intermediate (r.t. 23.1 min, method A) formed in proportion yields as high as 60% and was identified as the NCA **3** by a retention time identical to the authentic product and by HPLC-ESI-MS (negative mode [M–H] = 220.07, r.t. 1.96 min, method C). This intermediate was rapidly converted into the product **5** accompanied by the dipeptide H-Tyr(Me)-Tyr(Me)-OH. The presence of the dipeptide was confirmed by HPLC-ESI-MS (positive mode [M + H] = 373.2) as well as that of higher oligomers H-[Tyr(Me)]_n_-OH (with n = 3 to 5, [M + H] = 550.2, 727.3, 904.4 for peaks at r.t. 1.76 min, 1.93 min, 2.06 min, respectively, method C). By contrast reduced amounts of diketopiperazine *cyclo*-Tyr(Me)-Tyr(Me) formed confirming that the starting material lifetime was not sufficient for it to behave as a polymerization initiator leading to a dipeptide mixed anhydride prone to cyclization^27^. Under these conditions involving the presence of HCO_3_^−^, the polymerization into peptides thus proceeds through the NCA rather than directly from the starting material. An NCA intermediate was also observed to form rapidly at pH 7.5 in 100 mM MOPS buffers in the presence of added HCO_3_^−^ (Supplementary Information, Fig. S1). This behaviour indicates that the formation of long peptides from adenylates reported in the literature^9^,^10^ results probably from the polymerization of NCAs rather than from that of adenylates. The conversion of aminoacyl adenylates into NCA in the presence of CO_2_/HCO_3_^−^ was investigated starting from the Tyr(Me) derivative **1c** (Supplementary Information, Fig. S2). The conversion of **1c** into NCA was observed to proceed with rates similar to that observed for mixed anhydride **1b**. The release of AMP (r.t. 1.5 min, method A) accompanying the formation of NCA **3** could be detected by HPLC allowing the reaction to be monitored at 50 μM concentrations of reactant **1c** (r.t. 6.8 min, method A). The lifetime of the adenylate decreased with increasing concentrations of CO_2_/HCO_3_^−^ (*t*_1/2_ ~ 80 min, ~25 min, and <2 min at pH 6.5 in N_2_-flushed buffer, air equilibrated buffer and in the presence of 500 μM HCO_3_^−^, respectively). At pH 7.5 the lifetime of adenylate **1c** was reduced to less than 1 min in the presence of 500 μM HCO_3_^−^, which means that this mixed anhydride is likely to be converted into NCA within a few seconds at concentrations of CO_2_/HCO_3_^−^ above 2 mM and at pH value close to neutrality, which are representative of the present day ocean or physiological fluids. It is worth noting that this lifetime is not sufficient for peptides to be significantly formed by a direct reaction with adenylate so that any observation of peptide products under these conditions results for the most part from the intermediacy of NCAs.

**Figure 3:**
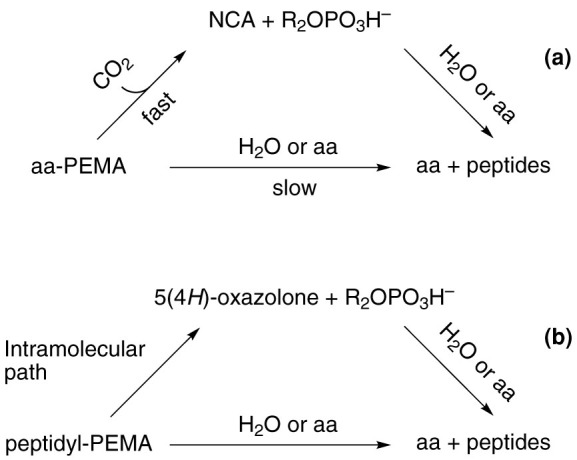
Intramolecular pathways competing with direct nucleophilic reactions for the conversion of mixed phosphate anhydrides. (a) The efficient hydrolytic pathway and conversion into peptides of amino acid phosphate ester mixed anhydrides promoted by carbon dioxide through NCAs. (b) Cyclization into 5(4*H*)-oxazolone competing with direct nucleophilic reaction of acyl-aa-PEMA and peptidyl-PEMA.

**Figure 4:**
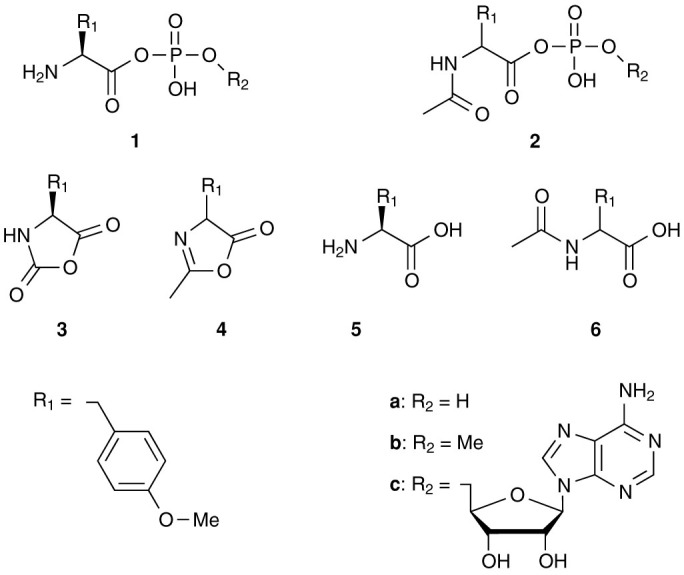
Structure of the reactants and products related to *O*-methyl-tyrosine (H-Tyr(Me)-OH) 5 studied in this work.

**Figure 5:**
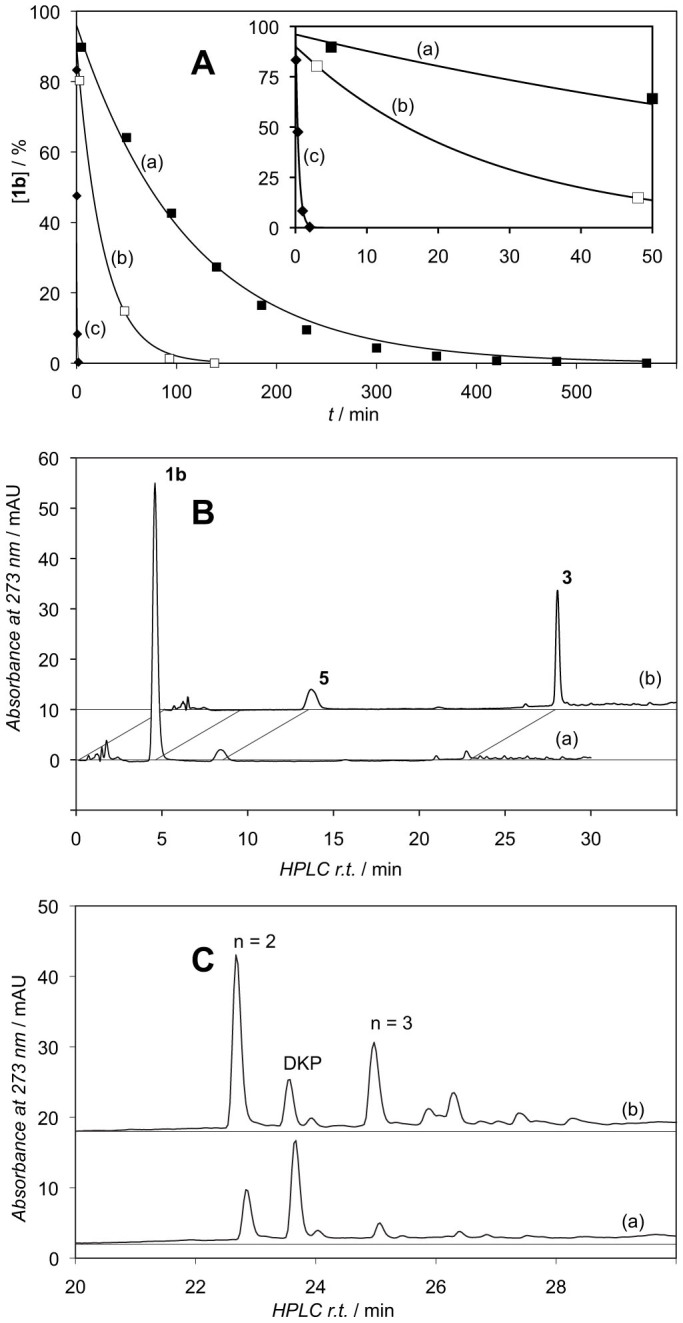
Hydrolysis of aa-PEMA 1b. Panel A - Monitoring by the evolution of its HPLC peak area (%, method A): (a) in a 100 mM pH 6.5 MES buffer flushed with N_2_ for 60 min (half-life c.a. 80 min, filled squares); (b) in a similar buffer equilibrated with air (half-life c.a. 18 min, open squares); (c) in a similar buffer to which was added 10 mM NaHCO_3_ (half-life c.a. 0.3 min, filled diamonds, 1 mL samples were withdrawn and acidified with 20 μL of 2 M formic acid before analysis); the expanded time scale in the inset shows that reaction (c) is completed within 2 min. Panel B – HPLC traces (method A) of samples withdrawn at 3 min from experiments carried out (a) in a 100 mM pH 6.5 MES buffer flushed with N_2_ for 60 min indicating the presence of unreacted mixed anhydride **1b** and a minor conversion to α-amino acid **5** and (b) in a 100 mM pH 6.5 MES buffer to which was added 2 mM NaHCO_3_ demonstrating a complete conversion of the starting material into NCA **3**. Panel C – HPLC traces corresponding to the range of retention times of the peptide products (method A, H-(Tyr(Me))_n_-OH with n = 2 to 5, r.t. 22.7, 24.9 min for n = 2, 3, respectively and diketopiperazine, *cyclo*-Tyr(Me)-Tyr(Me), DKP, r.t. 23.6 min) of the reactions after completion (a) in a 100 mM pH 6.5 MES buffer flushed with N_2_ for 60 min and (b) in a 100 mM pH 6.5 MES buffer to which was added 10 mM NaHCO_3_. HPLC peaks were identified by both injections of authentic samples and HPLC-ESI-MS analyses (method C) from similar experiments.

**Figure 6:**
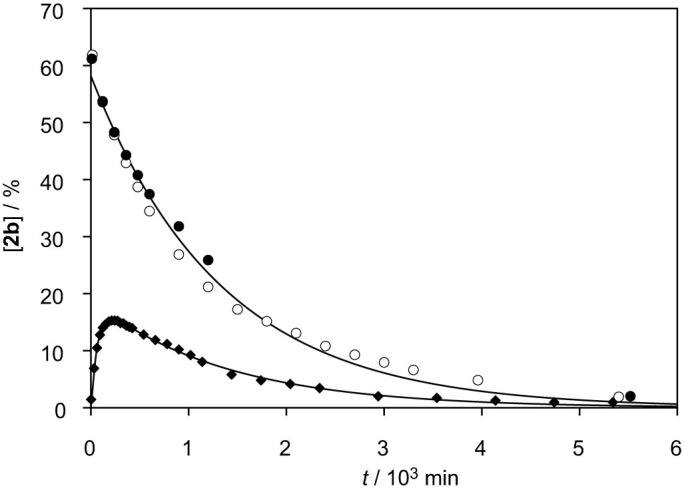
The hydrolysis of acyl-aa-PEMA 2b compared with the behaviour of the intermediate formed from the independent reaction of 5(4*H*)-oxazolone 4 with methyl phosphate. Evolution of **2b** monitored by the evolution of HPLC peak areas (%, method B) in several experiments. (a) Reaction of acyl-aa-PEMA **2b** in a 100 mM pH 6.5 MES buffer (*t*_1/2_ = c.a. 950 min, open circles) and (b) in a similar buffer to which 10 mM NaHCO_3_ was added (filled circles). (c) Formation (*t*_1/2_ = c.a. 50 min) and hydrolysis (*t*_1/2_ = c.a. 950 min) of mixed anhydride **2b** from 1 mM 5(4*H*)-oxazolone **4** in a 50 mM methyl phosphate buffer at pH 6.5 (filled diamonds).

**Figure 7:**
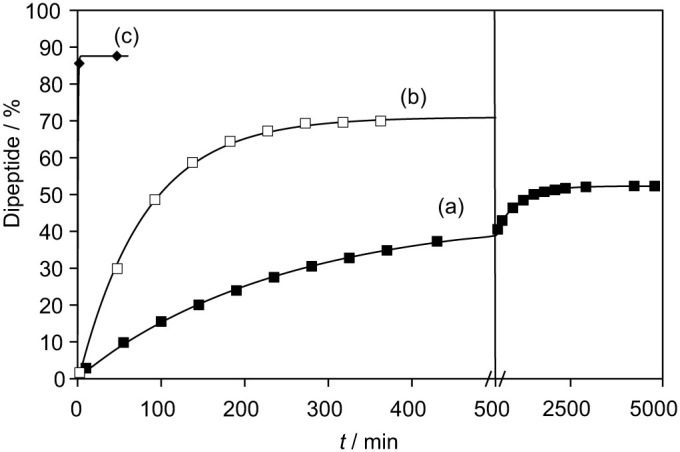
Formation of the dipeptides Ac-Tyr(Me)-Gly-NH_2_ or H-Tyr(Me)-Gly-NH_2_ by reaction of 5 mM H-Gly-NH_2_ with mixed anhydrides 2b or 1b, respectively, in 100 mM pH 6.5 MES buffers. Ratio of the peak area of formed peptides (%): (a) Reaction of 1 mM acyl-aa-PEMA **2b** (half-life c.a. 220 min, method A, r.t. 11.43 min, filled squares); (b) Reaction of aa-PEMA **1b** in the N_2_-flushed MES buffer (half-life c.a. 55 min, method A, r.t. 5.85 min, open squares); (c) Reaction of aa-PEMA **1b** in the MES buffer to which 2 mM NaHCO_3_ was added (half-life ≤1 min, method A, r.t. 5.85 min, filled diamonds). The peptide products were identified by ESI-MS.

At pH 4, the hydrolysis of mixed anhydride **1b** was much slower (*t*_1/2_ = ca. 550 min) and CO_2_ catalysis was not observed (Supplementary Information, Fig. S3). This result is consistent with the results obtained by Kluger from alanyl ethyl phosphate^24^. The protonation of the amino group of **1b** increases the electrophilic character of its acyl group and then the rates of nucleophilic attack, but it also prevents any possibility of reaction with CO_2_ according the pathway of Fig. 3a. The hydrolysis of the acetylated mixed anhydride **2b** was indeed observed to be slower (t_1/2_ ~ 950 min at pH 6.5) and was not affected by addition of 10 mM NaHCO_3_ (Fig. 6) in a way consistent with this explanation and with previously reported analyses^22^. However, it is important to emphasize that the CO_2_-catalyzed pathway does not only constitute a process leading to the deactivation and the hydrolysis of mixed anhydrides since peptide formation can be improved significantly by this means. As a matter of fact, with regard to peptide formation, the prevalence of the NCA pathway was demonstrated by studying the model reaction of 1 mM mixed anhydride **1b** with 5 mM glycinamide either in a nitrogen-flushed sample or in the presence of 2 mM NaHCO_3_ (Fig. 7). Importantly, less than 2 min were sufficient for the starting material to be exhausted in the presence of carbonate, whereas CO_2_ removal increased the reaction times to much higher values (*t*_1/2_ ~ 50 min) and reduced the final yield in dipeptide (Fig. 7). This reaction remained faster than that observed for the acetylated mixed anhydride **2b** (*t*_1/2_ ~ 260 min) unable to undergo the conversion into NCA, but that will be demonstrated below to partly undergo cyclization into 5(4*H*)-oxazolones. These experiments carried out using glycinamide for mimicking a growing peptide chain show that the polymerization of adenylates and other aa-PEMA is improved in the presence of CO_2_ by the occurrence of the NCA pathway owing to both the higher reactivity of the latter intermediate and its ability to suppress diketopiperazine formation.

### The interconversion of 5(*4H*)-oxazolones and acyl-aa-PEMA and peptidyl-PEMA

The reaction of Ac-Tyr(Me)-OH-derived oxazolone **4** in methyl phosphate-buffered aqueous solution (pH 6.5) at 20°C was monitored by HPLC and compared with the hydrolysis of mixed anhydride **2b** in MES buffers (Fig. 6). Comparable rates were observed and the intermediate of the 5(4*H*)-oxazolone **4** reaction was identified *in situ* by HPLC-ESI-HRMS (negative mode, calcd for C_13_H_17_NO_7_P^−^, 330.0743; found 330.0747) as the mixed anhydride **2b**. A similar behaviour was observed from a reaction of inorganic phosphate (Supplementary Information, Fig. S5). The hydrolysis of mixed anhydride **2b** was monitored by HPLC at 20°C in buffered solutions (Fig. 6). The reaction was also carried out in D_2_O to detect any hydrogen/deuterium exchange resulting from the transient formation of 5(4*H*)-oxazolone^20^,^28^ and compared to the product of a similar reaction of pure oxazolone **4** (Table 1). The values obtained demonstrate the occurrence of an intramolecular pathway already suspected from the higher rate of conversion of acylated aa-AMPs compared to simple acyl-adenylates^29^. At pH values below 5, the hydrolysis of anhydride **2b** (Supplementary Information, Fig. S4) has been observed to become faster in a way similar to the observation made by Lacey's group for Ac-Phe-AMP^22^. The identification of an intramolecular pathway made in the present work strongly suggests that the acid catalysis of acyl-aa-PEMA hydrolysis is the consequence of a facilitated cyclization from a good neutral phosphate leaving group. However, the absence of H/D exchange from the reaction of neither acyl-aa-PEMA **2b** nor 5(4*H*)-oxazolone **4** at this pH (Table 1) prevented any determination of the actual pathway of hydrolysis of mixed anhydride **2b**.

Similarly, we analyzed the degree of D/H exchange during the reaction of **2b** with L-Ala-NH_2_ in D_2_O at pH 6.5 (Table 1). The observation of a partial deuteration of the two diastereoisomers of the dipeptide product demonstrates that even when a better nucleophile is present, the α-proton is exchanged to a significant extent before the subsequent reaction of the 5(4*H*)-oxazolone takes place. The fast reaction of acyl-aa-AMP^29^ and other acyl-aa-PEMA results therefore, at least for a noticeable part, from a transient conversion into 5(4*H*)-oxazolones. Interestingly, the different degrees of deuteration of the two diastereomers indicate that the intramolecular path of Fig. 3b has a higher stereoselectivity as compared to the direct path (the reactants **2b** and **4** were prepared under a racemic form^28^).

## Discussion

As regards aa-PEMA reactions, it is noteworthy that CO_2_ catalysis proceeds through a pathway involving induced intramolecularity^30^. This kind of process shares one of the most important components of enzymatic activity, which corresponds to the utilization of binding energy to non-reacting portions of the substrate to bring about catalysis^31^. It was also proposed to constitute the easiest path for enzyme evolution under the name of *uniform binding*^32^ and is moreover necessary for enzymes to exceed a physical limit^33^. Induced intramolecularity has also been used to drive highly stereoselective catalysis in organic synthesis^34^,^35^. The efficiency of this kind of catalysis relies on the rates of intramolecular reactions^36^. Carbon dioxide present at total concentrations of ca. 30–40 μM in pH 6.5 solutions equilibrated with air (as deduced from the Henry's coefficient of CO_2_^37^ and the p*K*_a_ of carbonic acid) brings about a rate increase sufficient to render the catalytic pathway largely predominating, which is remarkable by considering a simple three-atom molecule compared to the efficiency of enzymes^38^. The ease of formation of 5-membered cycles from α-amino acid mixed anhydrides is also demonstrated by the conversion of acyl-aa-PEMA into 5(4*H*)-oxazolones.

These experiments demonstrating that the NCA path is prevailing at pH values close to neutrality in solutions equilibrated with air at present atmospheric levels of CO_2_ (*ca.* 0.04%) suggest that the pathway must be overwhelming in natural environments with higher contents. The experiments at 2 mM HCO_3_^−^ are representative of present day ocean total concentration of dissolved carbonate^39^ showing that the lifetime of aa-PEMA is expressed in tens of seconds in these media at pH 7.5. In biological media, with total carbonate concentrations approaching or exceeding 10 mM, the lifetime of mixed anhydrides would be even shorter. The early atmosphere had a CO_2_ content that remains poorly constrained^40^ but values similar to the present atmospheric levels^41^, or representing up to hundred times this value^40^,^42^, are often considered. Under these conditions, aa-PEMAs would be rapidly converted into NCA before any direct conversion into peptides could take place, which discards the earlier proposed contribution of aa-AMPs in the formation of prebiotic peptides^7^,^8^,^9^,^10^,^11^. Moreover, a less efficient polymerization ability of aa-PEMA and the diketopiperazine side-reaction make them improbable peptide precursors. The possibility that a very low content of CO_2_ in the atmosphere could have transiently permitted mixed anhydrides to be stabilized^23^ is made unlikely because it would have also required a very efficient removal of the most part of CO_2_ in the whole ocean (≥2 mM in HCO_3_^−^). On the contrary, the development of the activation pathway leading to translation must have occurred in an environment in which the role of NCA was unavoidable rather than in a local environment in which the mixed anhydrides were preserved from the presence of CO_2_ and HCO_3_^−^ by any kind of geochemical processes. NCA can be considered not only as intermediates of the degradation pathway of adenylates but also as precursors of any kind of aa-PEMA mixed anhydrides including adenylates as well as precursors of peptides through a pathway suppressing diketopiperazine side-reaction. From this point of view, the catalysis by carbon dioxide may lead to a fast exchange among different energy-rich species capable of linking activated amino acids to phosphorylating species. This distribution of energy in a reaction network, that may have anticipated the role of ATP as an energy currency, ensured a global far from equilibrium situation that was essential even at early stages of chemical evolution^43^. Considered from the point of view of a co-evolutionary development of peptide and nucleotide chemistries^44^ the CO_2_-catalyzed pathway may then constitute a key-element in the systemic integration of the two sub-systems^45^.

The fast conversion of adenylates, and more generally mixed anhydrides aa-PEMAs, into NCAs at low concentrations of CO_2_ in water questions the way through which the biochemical amino acid activation evolved. As a matter of fact, aa-AMPs, possibly produced from ATP through ribozyme activity^46^, would rapidly be converted into NCAs impeding the evolution of translation. Conversely, the catalytic activity of aaRSs might have evolved by acting on the thermodynamically favourable reverse reaction of aa-AMPs (formed spontaneously from NCAs) as a primitive pathway to produce ATP^2^,^3^. One could argue that the NCA pathway of Fig. 3a is still active in living cells but this speculation is not supported by any experimental data. However, the mechanism of pretransfer editing of misactivated aaRSs (through which adenylates are hydrolyzed) remains uncertain^47^. Any possible release of adenylates from the active site to solution^48^ during this step would lead to the formation of the corresponding NCA within seconds. Whatever NCA is actually or not a biochemical metabolite, the present results indicate that living organisms probably had to limit the importance of the release of adenylates into solution after translation evolved since a conversion into NCA would certainly lead to random aminoacylation of pending amino groups likely to be harmful to protein functional integrity. From this point of view, the *N*-formylation of methionine needed to initiate ribosomal peptide synthesis in bacteria might be considered as a remnant of a period in which NCA could be released in the cytoplasm. Therefore, we conclude that the potential formation of NCAs at least influenced the development of the translation apparatus and that of the aaRS family of enzymes in order to avoid random aminoacylation and that the NCA pathway must be taken into account in evolutionary studies.

Our analyses confirm the observations made by Lacey that CO_2_ is a very efficient catalyst for the conversion of adenylates. However, taking into account the probable role of NCAs and the diversity of processes made available through their intermediacy leads us to the very different conclusion that the process could be favourable to the development and evolution of life rather than solely detrimental to the role of adenylates as intermediates of peptide formation. It is also worth noting that acyl-aa-PEMA that were considered by Lacey as blocked equivalents of aa-AMPs^22^,^23^ does actually not constitute models of the reactivity of their parent compounds since they also undergo a spontaneous cyclization into 5(4*H*)-oxazolone. The transient formation of 5(4*H*)-oxazolone intermediates may be responsible for their efficiency in peptide formation^20^. The mixed anhydrides formed from free amino acids as well as peptide segments turn out to constitute unlikely precursors of peptides since their reactions are actually preceded by a very efficient cyclization into uncharged intermediates that thus constitute better electrophilic agents. This observation can be related to the evolutionary advantage of phosphate derivatives^49^ that is partly related to their negative charge reducing spontaneous hydrolytic degradation with respect to their enzyme-promoted reactions. From this perspective, their involvement required specific and efficient catalysts. However, the fact that NCA and 5(4*H*)-oxazolone also constitute precursors of mixed anhydrides through spontaneous processes provides a potential path through which these intermediates may have led for example to aminoacyl esters of RNA at predisposed locations^16^,^23^,^50^.

## Methods

Reagents and solvents were purchased from Bachem, Sigma-Aldrich, or Euriso-Top and used without further purification. Starting materials and products samples were prepared according to standard procedures and characterized by ^1^H, ^13^C and ^31^P NMR spectrometry and HRMS (Supplementary Information). NMR analyses were performed on a Bruker Avance 300 apparatus. HPLC analyses were performed on a Waters Alliance 2690 system with a photodiode array detector 996 using a Thermo Scientific BDS Hypersil C18 5 μm 2.1 × 50 mm column; mobile phase: A: H_2_O + 0.1% TFA, B:CH_3_CN + 0.1% TFA; flow rate: 0.2 mL/min and two different gradients; method A: 0 min (5% B), to 15 min (15% B), 25 min (60% B) and 26 min (100% B); method B: 0 min (5% B), to 10 min (20% B), 11 min (100% B). HPLC-ESI-MS analyses were carried out on a Waters Synapt G2-S system connected to a Waters Acquity UPLC H-Class apparatus equipped with a Acquity UPLC BEH C18, 1.7 μm 2.1 × 50 mm column; method C: A: H_2_O + 0.01% formic acid, B: acetonitrile + 0.01% formic acid; flow rate: 0.5 mL/min; linear gradient 0% to 100% B over 3 min.

## Author Contributions

Z.L., J.-C.R. and R.P. designed research; Z.L. performed research; D.B. contributed new reagents and analytic tools; Z.L., J.-C.R. and R.P. analyzed data and wrote the paper.

## Supplementary Material

Supplementary Information SUPPLEMENTARY INFORMATION

## Figures and Tables

**Figure 1:**
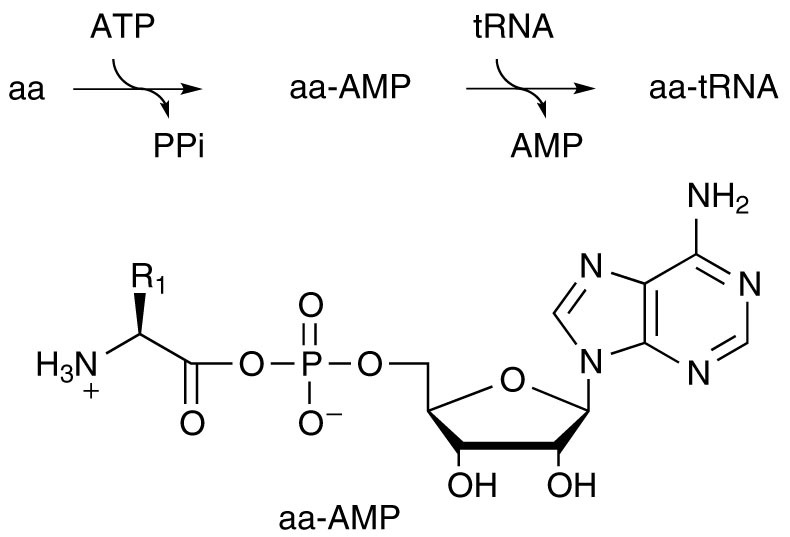
The aaRS-catalyzed reaction of α-amino acids with ATP.

**Figure 2:**
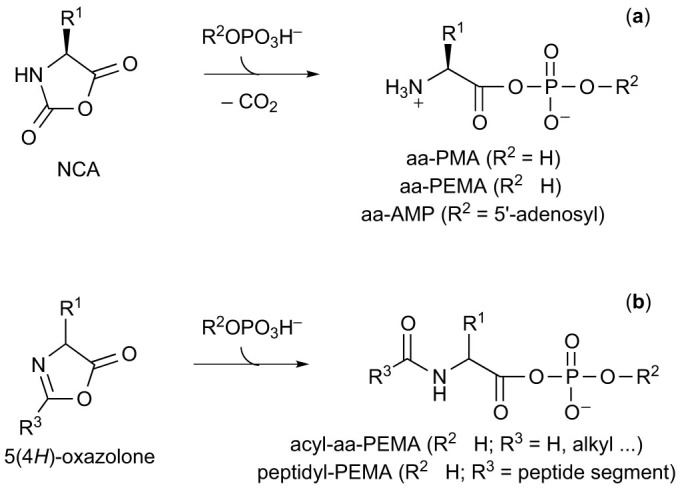
Potential pathways for the abiotic formation of mixed anhydrides of α-amino acids and peptides with phosphate (PMA) and phosphate esters (PEMA) including adenylates (AMP). (a) Reaction of NCAs with phosphate esters; (b) The hypothesized similar reaction of 5(4*H*)-oxazolones.

**Table 1:** Results of H/D exchange experiments carried out from either acetylated mixed anhydride 2b or oxazolone 4 under various conditions

		Ratio of [M + 1] MS peak in the product corrected from natural abundance^a^	
Nucleophile	Buffer	Mixed anhydride 2b	Oxazolone 4
H_2_O	100 mM formate^b^	<1%	<1%
H_2_O	100 mM MES^c^	55%	≥93%
H-Ala-NH_2_	100 mM MES^c^	27% (L, L-isomer)	99% (L, L-isomer)
		16% (D, L-isomer)	98% (D, L-isomer)

^a^Determination by ESI-MS: after the reaction in D_2_O the residue was freeze-dried and then submitted three times to cycles of addition of H_2_O and freeze-drying to convert any easily exchangeable hydrons and finally analyzed by MS;

^b^Buffer ratio equivalent to that of a pH 4 buffer in H_2_O;

^c^buffer ratio equivalent to that of a pH 6.5 buffer in H_2_O.
