# Supplementary material for: Evolutionary Importance of the Intramolecular Pathways of Hydrolysis of Phosphate Ester Mixed Anhydrides with Amino Acids and Peptides
Source: Sci Rep. 2014 Dec 11;4:7440. doi: 10.1038/srep07440 (PMC4262824; doi:10.1038/srep07440)
Supplement: Supplementary Information [file srep07440-s1.pdf]

SUPPLEMENTARY INFORMATION

Evolutionary Importance of the Intramolecular Pathways of Hydrolysis of Phosphate Ester Mixed Anhydrides with Amino Acids and Peptides

Ziwei Liu, Damien Beaufils, Jean-Christophe Rossi & Robert Pascal

Experimental details for the synthesis of chemicals 2

N-t-Butyoxycarbonyl-O-methyl-tyrosine, Boc-Tyr(Me)-OMe 2

Acetyl-*O*-methyl-tyrosine, Ac-Tyr(Me)-OH, **6**. 2

2-Methyl-4(4-methoxybenzyl)-5(4*H*)oxazolone, **4** 3

4(4-methoxy)benzyl-oxazolidin-2,5-dione, Tyr(Me)-NCA, **3** 3

Methylphosphate, bis(cyclohexylammonium) salt 3

N-Acetyl-O-methyl-D,L-tyrosyl-(methyl)phosphate, diisopropylethylamine salt, **2b**·DIEA 4

O-Methyl-tyrosyl-(methyl)phosphate, diisopropylethylamine salt, **1b**·DIEA 4

O-Methyl-tyrosyl-5'-adenylate, **1c** 4

Boc-Tyr(Me)-Tyr(Me)-OMe 5

H-Tyr(Me)-Tyr(Me)-OH 5

Cyclo(Tyr(Me)-Tyr(Me)) 5

Experimental details for conducting hydrolysis and aminolysis reactions 6

Slow reactions. 6

Fast reactions of mixed anhydrides **1b** and **1c** in the presence of bicarbonate 6

References 6

Supplementary figures 8

Experimental details for the synthesis of chemicals

N-t-Butyoxycarbonyl-O-methyl-tyrosine, Boc-Tyr(Me)-OMe

The title compound was prepared by a procedure described in the literature[S1,S2]. A solution of Boc-Tyr-OH (7.0 mmol, 2.0 g) in DMF (20 mL) was cooled using an ice bath, treated with freshly ground KOH (7.7 mmol, 0.43 g), and a cooled solution of CH_3I (7.7 mmol, 0.49 mL) in DMF (5 mL) was added dropwise over 1 min. The mixture was stirred at room temperature for 30 min, cooled using an ice bath, and additional KOH (7.7 mmol, 0.43 g) and a cooled solution of CH_3I (7.7 mmol, 0.49 mL) in DMF (5 mL) were added dropwise over 1 min. The mixture was stirred for 3 h, poured onto ice (40 g), and extracted with ethyl acetate (3 × 20 mL). The organic layers were washed with water (3 × 13 mL), brine (2 × 13 mL), and dried over $NaSO_4$. The solvent was removed under reduced pressure to afford a colourless oily residue (yield 1.33 g, 60.5%). Then the oil was purified by preparative silica gel chromatography on (mobile phase: ethyl acetate - light petroleum, 3:7 *v*/*v*). ^{1}H NMR (300 MHz, $CDCl_3$) δ ppm 7.04 (d, *J* = 8.59 Hz, 2H), 6.90-6.79 (m, 2H), 4.54 (dd, *J* = 13.43, 5.86 Hz, 1H), 4.13 (q, *J* = 7.14 Hz, 1H), 3.79 (s, 3H), 3.72 (s, 3H), 3.03 (t, *J* = 5.79 Hz, 2H), 1.42 (s, 9H).

Acetyl-*O*-methyl-tyrosine, Ac-Tyr(Me)-OH, 6.

This compound was prepared in two steps. In a first stage, Ac-Tyr-OH was methylated using a method similar to that used previously for Boc-Tyr-OH. To this aim, a solution of Ac-Tyr-OH (20 mmol, 4.46 g) in DMF (56 mL) was cooled using an ice bath, treated with freshly ground KOH (22 mmol, 1.20 g), and a cooled solution of CH_3I (22 mmol, 1.37 mL) in DMF (14 mL) was then added dropwise over 3 min. The mixture was stirred at room temperature for 2 h, cooled using an ice bath, and additional KOH (22 mmol, 1.20 g) and a cooled solution of CH_3I (22 mmol, 1.37 mL) in DMF (14 mL) were added dropwise over 3 min. The mixture was stirred for 16 h, poured onto ice (150 g), and extracted with ethyl acetate (3 × 75 mL). The organic layers were washed with water (3 × 50 mL), brine (2 × 50 mL), and dried by $NaSO_4$. The solvent was removed under reduced pressure to afford a colourless oily residue. Then crystallization was achieved from ethyl acetate/light petroleum, to give Ac-Tyr(Me)-OMe as colourless crystals (yield 3.14 g, 62.5%). ^{1}H NMR (300 MHz, *DMSO*) δ ppm 8.30 (d, *J* = 7.73 Hz, 1H), 7.25-7.00 (m, 2H), 6.97-6.72 (m, 2H), 4.39 (ddd, *J* = 9.01, 7.83, 5.71 Hz, 1H), 3.72 (s, 3H), 3.59 (s, 3H), 2.87 (ddd, *J* = 22.92, 13.80, 7.41 Hz, 2H), 1.80 (d, *J* = 5.10 Hz, 3H); ^{13}C NMR (*DMSO*) δ ppm 172.74 (s), 169.77 (s), 158.43 (s), 130.50 (s), 129.52 (s), 114.12 (s), 55.42 (s), 54.35 (s), 52.23 (s), 36.41 (s), 22.72 (s).

Ac-Tyr(Me)-OMe (5 mmol, 1.23 g) was then dissolved in iPrOH:H_2O (7:3 *v*/*v*) (minimum volume), treated with NaOH (6 mmol, 0.24 g). The mixture was stirred at room temperature for 2 h. iPrOH was removed under reduced pressure to afford a solid residue. The residue was dissolved in water (10 mL) and passed through a column of Dowex 50 ion-exchange resin (H^+ form) to remove sodium. Then the aqueous solution was freeze-dryed. The residue was crystallized from ethyl acetate/light petroleum, to give Ac-Tyr(Me)-OH under the form of colourless crystals (yield 0.98 g, 84.5%). ^{1}H NMR (300 MHz, *DMSO*) δ ppm 8.14 (d, *J* = 8.04 Hz, 1H), 7.14 (d, *J* = 8.46 Hz, 2H), 6.84 (d, *J* = 8.48 Hz, 2H), 4.34 (dt, *J* = 8.96, 5.02 Hz, 1H),

3.72 (s, 3H), 2.86 (ddd, J = 23.23, 13.83, 7.18 Hz, 2H), 1.78 (s, 3H). HRMS (ESI positive mode): calcd for $C_{12}H_{15}NNaO_4^+$, 260.0899; found 260.0896.

2-Methyl-4(4-methoxybenzyl)-5(4*H*)oxazolone, 4

A solution of Ac-Tyr(Me)-OH (2.11 mmol, 500 mg) in CH_2Cl_2 (20 mL) was cooled using an ice bath, treated with EDC (2.87 mmol, 445 mg). The mixture was maintained at 0 °C for 1 h under stirring. Then CH_2Cl_2 (20 mL) was added. The solution was washed with H_2O (2 × 20 mL), a saturated aqueous solution of $NaHCO_3$ (1 × 20 mL), brine (1 × 20 mL), and dried with Na_2SO_4. The solvent was removed under vacuum to afford a colourless oily residue (yield 440 mg, 95.2%). The product was allowed to crystallize at +4°C. As determined earlier, this procedure result in a loss of chirality and oxazolone **4** is obtained as a racemic mixture.[28] ^{1}H NMR (300 MHz, DMSO) δ ppm 7.20-7.03 (m, 2H), 6.91-6.78 (m, 2H), 4.63 (tq, J = 4.16, 2.04 Hz, 1H), 3.72 (s, 3H), 2.97 (ddd, J = 21.29, 14.11, 6.11 Hz, 2H), 2.06 (d, J = 2.08 Hz, 3H); 13C NMR (*DMSO*) δ ppm 178.83 (s), 162.12 (s), 158.58 (s), 130.85 (s), 128.33 (s), 113.98 (s), 65.97 (s), 55.41 (s), 35.66 (s), 15.05 (s). HRMS (ESI negative mode): calcd for $C_{12}H_{12}NO_3^-$, 218.0817; found 218.0796

4(4-methoxy)benzyl-oxazolidin-2,5-dione, Tyr(Me)-NCA, 3

The NCA was prepared using a standard method[S3,S4]. A mixture of H-Tyr(Me)-OH (2.0 mmol, 390 mg) in anhydrous THF (4 mL) was heated to 50 °C using an oil bath, then triphosgene (0.67 mmol, 0.198 g) was added. The mixture was stirred for 20 h under a N_2 atmosphere. The unreacted starting material was removed by filtration after the mixture cooled to room temperature. The product was precipitated by addition of hexane (12 mL) and cooling at –20 °C. The product was collected by filtration. Yield 326 mg, 73.8%. ^{1}H NMR (300 MHz, acetone-d6) δ ppm 7.95 (s, 1H), 7.20 (d, J = 8.69 Hz, 2H), 6.89 (d, J = 8.72 Hz, 2H), 4.82 (ddd, J = 5.81, 4.85, 1.01 Hz, 1H), 3.78 (s, 3H), 3.13 (dq, J = 14.29, 5.30 Hz, 2H); ^{13}C NMR (acetone-d6) δ ppm 170.36 (s), 159.09 (s), 151.60 (s), 130.82 (s), 126.62 (s), 113.89 (s), 58.87 (s), 54.59 (s), 36.02 (s); HRMS (ESI negative mode): calcd for $C_{11}H_{10}NO_4$, 220.0610; found 220.0613.

Methylphosphate, bis(cyclohexylammonium) salt

Methyl phosphate was prepared through a published procedure[S5]. Pure anhydrous phosphoric acid (20.4 mmol, 2.0 g) was introduced in a solution of pyridine (102 mmol, 8.3 mL), methanol (204 mmol, 8.24mL), and triethylamine (40.8 mmol, 5.6 mL). Acetic anhydride (40.8 mmol, 3.86 mL) was added to the mixture which was then heated to 90 °C and stirred for 20 h in an oil bath. After cooling to room temperature, water (10 mL) was added and the mixture was stirred at 90 °C for 3 h in an oil bath, poured onto water (24 mL), and washed by ethyl acetate (3×50 mL). Water was removed under reduced pressure to afford a colourless oil. The oil was dissolved in acetone/water (9:1, 100 mL), treated by cyclohexylamine (7 mL). The mixture was kept in 4 °C overnight. The solid residue was recovered by filtration and then dissolved in pure ethanol. The mixture was heated to the boiling point, the insoluble solid was removed by filtration while hot through filter paper. The solution was cooled and maintained at +4 °C until crystallization. The solid residue was filtered and dried (2.07 g, 35%). ^{1}H NMR (300 MHz, D_2O) δ ppm 3.38 (d, J = 10.12 Hz, 3H), 3.07 (dq, J = 11.16, 4.34 Hz, 2H); ^{31}P NMR (D_2O) δ ppm 4.67 (s).

N-Acetyl-O-methyl-D,L-tyrosyl-(methyl)phosphate, diisopropylethylamine salt, 2b·DIEA

Cyclohexylamine was exchanged for DIEA from methylphosphate, bis(cyclohexylammonium) salt (2 mmol, 0.62 g) dissolved in water (10 mL) using a Dowex 50 (H^+ form) ion exchange resin. The solution was introduced and the column was extensively eluted with water. The eluate was collected in a flask containing DIEA (2.2 mmol, 380 µl) and then freeze-dried. The residue was dissolved in CH_3CN (20 mL), cooled to 0°C and allowed to react with a solution of oxazolone **4** (2.2 mmol, 468.9 mg) in CH_3CN (20 mL). The mixture was stirred for 1.5 h in an ice bath. The solvent was removed under vacuum to afford a colourless oil. The oil was dissolved in CH_2Cl_2 (50 mL), extracted by H_2O (3×20 mL). The aqueous layer was collected, placed under vacuum to remove the residual organic solvent and then freeze-dried. Since the synthesis was performed[28] from a racemized oxazolone **4** the product was obtained under a racemic state. ^{31}P NMR (DMSO) δ ppm -8.25 (s); HRMS (ESI negative mode): calcd for $C_{13}H_{17}NO_7P^-$, 330.0748; found 330.0743.

O-Methyl-tyrosyl-(methyl)phosphate, diisopropylethylamine salt, 1b·DIEA

A solution of Boc-Tyr(Me)-OH (2.2 mmol, 0.65 g) in CH_2Cl_2 (20 mL) was activated by DCC (2.0 mmol, 0.413 g) at 0°C for 1.5 h. The resulting mixture was reacted with a freshly prepared batch of mono-methyl phosphate DIEA salt (2 mmol) dissolved in CH3CN (20 mL). After stirring 1.5 h at room temperature, DCU was removed by filtration. The solvent was removed under reduced pressure to afford a colourless oily residue. The residue was dissolved in acetone and purified by column chromatography on a short silica column using acetone-methanol (9:1 *v*/*v*) as a mobile phase (yield 0.82 g, 71.9%). Then the Boc-protected mixed anhydride (0.23 mmol, 0.12 g) was reacted in TFA (1 mL, 13.5 mmol) for 30 min. After addition of ether (5 mL), the solid was centrifugated and, after removing the solvent, it was washed repeatedly with ether (2 × 5 mL), CH_2Cl_2 (2 × 5 mL) and ether (1 × 5 mL), and dried under vacuum (yield 17.7 mg 26.4%). ^1H NMR (300 MHz, D_2O) δ ppm 7.21 (d, J = 8.62 Hz, 2H), 6.94 (d, J = 8.67 Hz, 2H), 3.75 (s, 3H), 3.54 (d, J = 11.61 Hz, 3H), 3.48 (q, J = 7.06 Hz, 1H), 3.21 (dq, J = 15.17, 14.97, 6.89 Hz, 2H); ^{31}P NMR (D_2O) δ ppm –6.25 (s); HRMS (ESI negative mode): calcd for $C_{11}H_{15}NO_6P^-$, 288.0637; found 288.0644.

O-Methyl-tyrosyl-5'-adenylate, 1c

The adenylate was prepared from the Boc-protected amino acid Boc-Tyr(Me)-OH[S6]. An aqueous solution of 5'-AMP (0.365 g, 1 mmol) was converted into a salt by adding DIEA (190 µl, 1.1 equiv). The solution was frozen and lyophilized. Independently, Boc-Tyr(Me)-OH (1.32 g, 4.4 mmol) was activated by reaction with DCC (0.828 g, 4 mmol) for 2 h in CH_2Cl_2 (10 mL) at room temperature. The N,N'-dicyclohexylurea by-product was removed by filtration and the solution was concentrated *in vacuo* without heating, the residue was diluted with DMF and added to the 5'-AMP-DIEA salt (1 mmol) with further DIEA (190 µl, 1.1 mmol). The reaction mixture was stirred overnight at room temperature and the product was precipitated by addition of Et_2O. The precipitate was collected by filtration. The solid was further washed with Et_2O and dried *in vacuo*. The resulting white solid residue was dissolved in TFA (1 mL) and allowed to react at room temperature for 30 min. Excess Et_2O was added and the precipitate was collected by centrifugation. The solid was further washed with Et_2O, twice with CH_2Cl_2 and again with Et_2O before being dried *in vacuo*. Then the solid was

purified by preparative reverse phase HPLC (C18 column, mobile phase: A: H_2O+0.1% TFA, B: CH_3CN+0.1% TFA; flow rate: 9 mL/min gradient: 0 min (5% B), to 14 min (25% B) and 19 min (100% B). ^1H NMR (300 MHz, D_2O) δ ppm 8.37 (s, 1H), 8.21 (s, 1H), 6.88 (d, *J* = 8.69 Hz, 2H), 6.69 (d, *J* = 8.70 Hz, 2H), 6.05 (d, *J* = 4.95 Hz, 1H), 4.62 (t, *J* = 5.08 Hz, 1H), 4.42 (t, *J* = 5.05 Hz, 1H), 4.27 (t, *J* = 6.28 Hz, 2H), 4.15 (dd, *J* = 5.89, 2.24 Hz, 2H), 3.64 (s, 3H), 2.81 (t, *J* = 5.83 Hz, 2H); ^{13}C NMR (D_2O) δ ppm 166.04 (s), 158.30 (s), 149.95 (s), 148.21 (s), 145.26 (s), 142.03 (s), 130.32 (s), 125.34 (s), 118.34 (s), 114.31 (s), 87.72 (s), 83.44 (s), 74.01 (s), 69.87 (s), 66.14 (s), 55.21 (s), 54.26 (s); ^{31}P NMR (D_2O) δ ppm -7.99 (s).

Boc-Tyr(Me)-Tyr(Me)-OMe

Boc-Tyr(Me)-OMe (1 g, 3.24 mmol) was treated by TFA to remove the Boc group. A solution of the TFA salt of H-Tyr(Me)-OMe (0.8 g, 2.48 mmol) and Boc-Tyr(Me)-OH (0.81 g, 2.613 mmol) in the minimum volume of DMF was cooled in an ice bath. The mixture was activated with PyBOP (1.36 g, 2.613 mmol) and DIEA (1 mL, 5.749 mmol, 2.2 eq). After stirring overnight, the mixture was poured onto ice (20 g). The white solid was collected by filtration and washed by $NaHCO_3$ saturated aqueous solution, $KHSO_4$ saturated aqueous solution and water, then dried under vacuum over P_2O_5 (yield 1.14 g, 83.2%). This intermediate was used for the subsequent preparation of the dipeptide samples needed for identification purpose. ^1H NMR (300 MHz, *DMSO*) δ ppm 8.25 (d, *J* = 7.57 Hz, 1H), 7.14 (d, *J* = 8.53 Hz, 4H), 6.84 (d, *J* = 8.53 Hz, 4H), 4.45 (dd, *J* = 13.76, 7.54 Hz, 1H), 4.12 (dt, *J* = 9.53, 4.22 Hz, 1H), 3.71 (s, 6H), 3.58 (s, 3H), 3.10-2.71 (m, 4H), 1.30 (s, 9H), 2.62 (dd, *J* = 13.66, 10.32 Hz, 1H).

H-Tyr(Me)-Tyr(Me)-OH

Boc-Tyr(Me)-Tyr(Me)-OMe (0.5 mmol, 243 mg) was dissolved in the minimum volume of a 7:3 mixture of iPrOH and H_2O, then 1M aqueous NaOH (0.75 mL) was added. The mixture was stirred at room temperature for 2 h. After the mixture was concentrated under reduced pressure, the resulting aqueous solution was acidified with acetic acid, the white solid was collected by filtration and dried. The solid was then treated by TFA/H_2O (9:1 *v*/*v*) for 30 min to remove the Boc-protecting group. TFA was removed under vacuum, water was added and the solution was extracted with ether, then the aqueous phase was concentrated and freeze-dried (yield 30 mg, 15.5%). ^1H NMR (300 MHz, D_2O) δ ppm 6.54 (dd, *J* = 14.69, 8.57 Hz, 4H), 6.29 (dd, *J* = 8.38, 5.82 Hz, 4H), 3.98 (dd, *J* = 7.71, 6.12 Hz, 1H), 3.59 (t, *J* = 7.13 Hz, 1H), 3.18 (d, *J* = 3.39 Hz, 6H), 2.44 (tt, *J* = 14.17, 6.26 Hz, 4H); ^{13}C NMR (D_2O) δ ppm 173.28 (s), 168.19 (s), 158.07 (s), 157.46 (s), 130.17 (s), 129.83 (s), 128.13 (s), 125.52 (s), 114.06 (s), 113.65 (s), 54.77 (s), 53.91 (s), 35.44 (s), 35.08 (s); HRMS (ESI negative mode): calcd for $C_{20}H_{25}N_2O_5^-$, 373.1763; found, 373.1764

Cyclo(Tyr(Me)-Tyr(Me))

Boc-Tyr(Me)-Tyr(Me)-OMe (0.5 mmol, 243 mg) was treated by TFA/H_2O (9:1 *v*/*v*) for 30 min. The TFA was removed under vacuum, ether was added in to precipitate the product. The solid was collected by filtration and dissolved in methanol and DIEA (0.87 mL, 5 mmol) was added. The mixture was stirred at 60 °C for 24 h. The mixture was cooled at room temperature and the solid was collected by filtration and washed by ether (yield 10 mg,

5.4%). ^{1}H NMR (300 MHz, DMSO) δ ppm 7.87 (s, 2H), 6.95 (d, *J* = 8.47 Hz, 4H), 6.85 (d, *J* = 8.50 Hz, 4H), 3.92 (s, 2H), 3.70 (s, 6H), 2.21 (dd, *J* = 13.61, 6.10 Hz, 2H); ^{13}C NMR (DMSO) δ ppm 166.72 (s), 158.51 (s), 131.27 (s), 128.85 (s), 114.14 (s), 56.04 (s), 55.48 (s); HRMS (ESI, positive mode): calcd for $C_{20}H_{23}N_2O_4^+$, 355.1658; found, 355.1661.

Experimental details for conducting hydrolysis and aminolysis reactions

Slow reactions.

Reactions in buffered media. Reactions were usually performed by monitoring the reaction progress in 1 mL samples of the reaction medium kept in the HPLC system located in a room maintained at 20°C. The reaction progress was monitored by the HPLC integral of peaks at 273 nm corresponding to the absorbance of the aryl ether moiety of the Tyr(Me) residue, making the assumption that reactions provoked no change in the absorbance of the chromophoric group, which is reasonable with respect to the limited precision required for the present study.

Reaction in degassed buffer. One of the following procedures was used: (a) Usually, CO_2 was removed from the reaction buffer by bubbling N_2 previously washed by passing through a 1N NaOH solution for 30 min or 60 min. Alternatively, (b), the following more radical procedure was used to monitor the rate of the highly CO_2-sensitive reaction of H-Tyr(Me)-OPO_3HMe **1b**. The 100 mM MES pH 6.5 buffer (5 mL) was treated by a freeze-pump-thaw degassing progress. The solution was placed in a Schlenk flask and the flask was sealed and then frozen in liquid acetone cooled at the melting point (–95°C). When the solution was frozen, the stopcock was open to vacuum line and the remaining gas was pumped for 5 min. The flask was sealed and the solution allowed to melt in a tepid water bath. The water bath was then replaced by a liquid-solid mixture of acetone. The whole procedure was repeated for 8 times. Then solid H-Tyr(Me)-OPO_3HMe **1b** (0.005 mmol, 1.45 mg) was dropped on the frozen solution. The flask was subsequently sealed and pumped to remove gases from the atmosphere. Then, the solution was melted, the substrate was dissolved under stirring and the medium was allowed to react at room temperature in the sealed flask. After 200 min, a 1 ml sample was withdrawn and analyzed by HPLC (HPLC Method A).

Fast reactions of mixed anhydrides 1b and 1c in the presence of bicarbonate

The fast reactions of mixed anhydrides **1b** and **1c** in the presence of $NaHCO_3$ were monitored by withdrawing 1 mL samples of the reaction medium that were acidified by adding 0.2 M formic acid (20 µl). The mixtures were stored in ice and rapidly analyzed by HPLC (HPLC method A).

References

S1 Bulman Page, P. C., Buckley, B. R., Rassias, G. A. & Blacker, A. J. New Chiral Iminium Salt Catalysts for Asymmetric Epoxidation. *Eur. J. Org. Chem.* 803–813 (2006).

S2 Buckley, B. R., Bulman Page, P. C. & McKee, V. A Rapid and Highly Diastereoselective Synthesis of Enantiomerically Pure (4*R*,5*R*)- and (4*S*,5*S*)-Isocytoxazone *Synlett* 1399–1402 (2011).

S3 Daly, W. H. & Poche, D. The preparation of *N*-carboxyanhydrides of amino acids using bis(trichloromethyl)carbonate. *Tetrahedron Lett.* **29**, 5859-5862 (1988).

S4 Kramer, J. R. & Deming, T. J. General Method for Purification of alpha-Amino acid-*N*-carboxyanhydrides Using Flash Chromatography. *Biomacromolecules* **11**, 3668–3672 (2010).

S5 Dueymes, C., Pirat, C. & Pascal, R. Facile synthesis of simple mono-alkyl phosphates from phosphoric acid and alcohols. *Tetrahedron Letters* **49,** 5300–5301 (2008).

S6 Armstrong, D. W., Seguin, R., Saburi, M. & Fendler, J. H. Synthesis of amino acyl adenylates using the tert-butoxycarbonyl protecting group. *J. Mol. Evol.* **13**, 103–113 (1979).

Supplementary figures

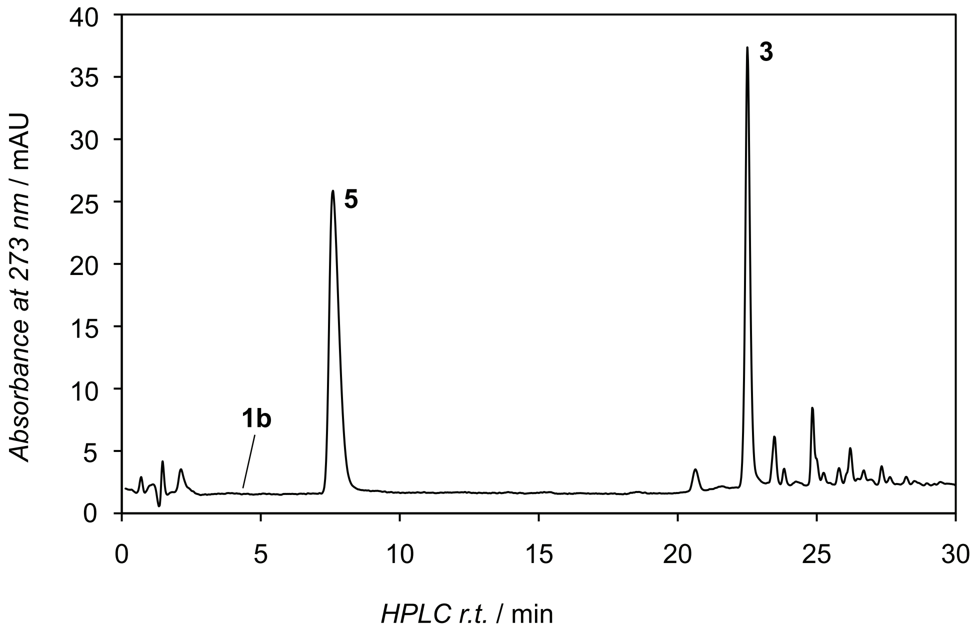

Fig. S1 | Intermediacy of NCA at pH 7.5. HPLC trace of a 1 mL sample of the reaction of 1 mM aa-PEMA **1b** in presence of 2 mM $NaHCO_3$ in 100 mM MOPS buffer (pH 7.5) withdrawn after 3 min (HPLC method A).

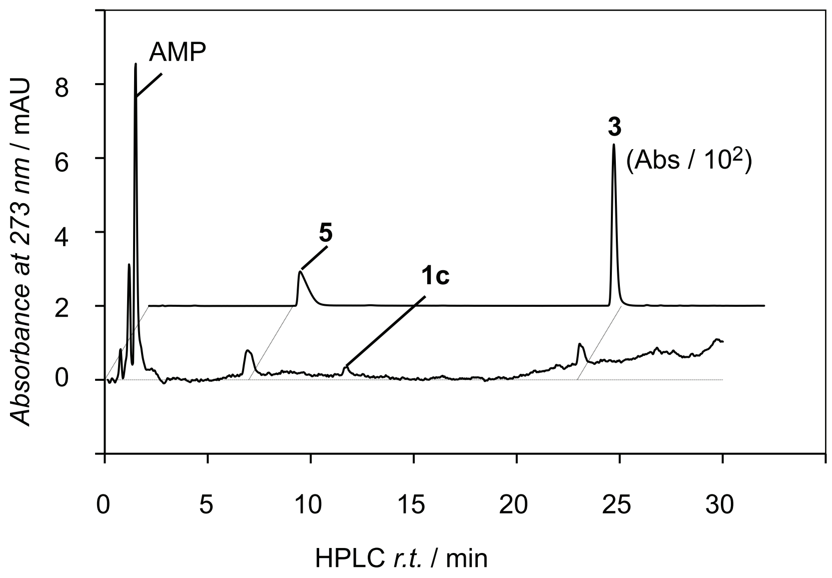

Fig. S2 | Hydrolysis of 50 µM Tyr(Me) adenylate 1c in a 100 mM MES buffer (pH 6.5). HPLC trace of a sample of the reaction medium withdrawn at 3 min. Comparison with a solution of synthetic sample of Tyr(Me)-NCA **3** in water acidified by 0.1% TFA (HPLC Method A).

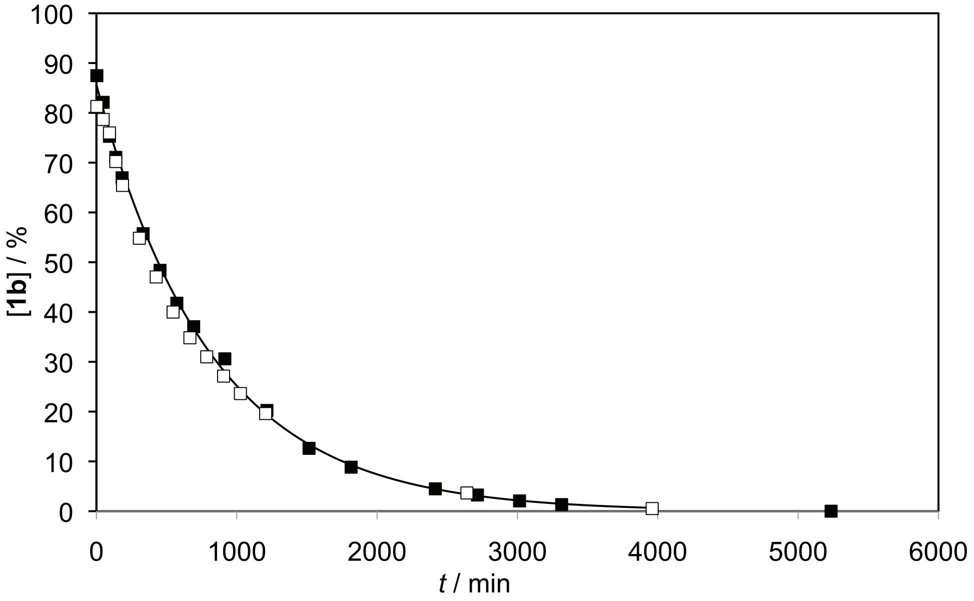

Fig. S3 | Hydrolysis of H-Tyr(Me)-OPO_3HMe 1b in a 100 mM formic acid buffer (pH 4). 1 mM aa-PEMA **1b** was dissolved in 1 mL pH 4 formic acid buffer (after removing CO_2 by flushing the solution with N_2 before the reaction, open squares, and without any attempt to remove CO_2, filled squares); analysis by HPLC (HPLC method A).

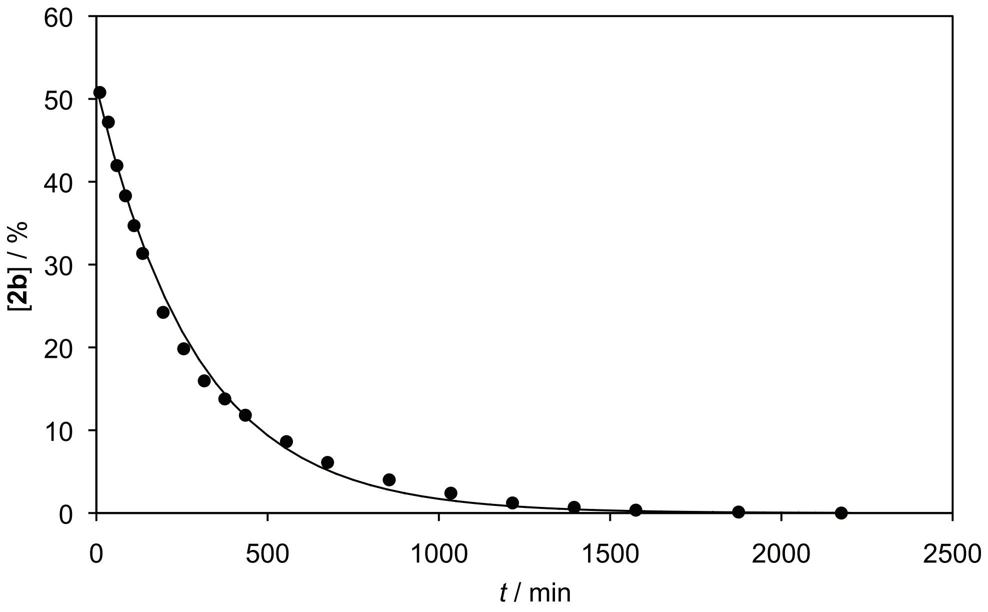

Fig. S4 | Hydrolysis of Ac-Tyr(Me)-OPO$_3$HMe 2b in a 100 mM formic acid buffer (pH 4). 1 mM acyl-aa-PEMA **2b** was dissolved in 1 mL buffer; analysis by HPLC (HPLC method B).

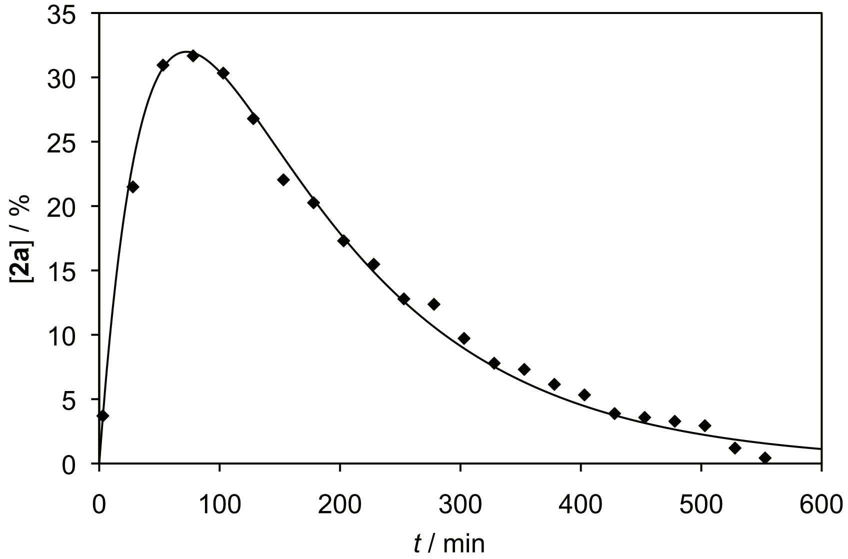

Fig. S5 | HPLC monitoring of acyl-aa-PEMA formation from the reaction of 1 mM 5(4*H*)-oxazolone 4a in 50 mM KH_2PO_4/NaOH buffer (pH 6.5). Analysis by HPLC (method B). The reaction was started by adding a 20 mM CH_3CN solution of Ac-Tyr(Me)-5(4H)-oxazolone (50 µl) in a 50 mM KH_2PO_4/NaOH (2:1) buffer (1 mL); analysis by HPLC (method A) and the yield of the intermediate (retention time 7.8 min) was determined by the area of HPLC peaks.
